# Breast density knowledge and willingness to delay treatment for pre-operative breast cancer imaging among women with a personal history of breast cancer

**DOI:** 10.1186/s13058-024-01820-x

**Published:** 2024-04-29

**Authors:** Rebecca E. Smith, Brian L. Sprague, Louise M. Henderson, Karla Kerlikowske, Diana L. Miglioretti, Karen J. Wernli, Tracy Onega, Roberta M. diFlorio-Alexander, Anna N.A. Tosteson

**Affiliations:** 1 The Dartmouth Institute for Health Policy and Clinical Practice, Geisel School of Medicine at Dartmouth College, 1 Medical Center Dr. WTRB Level 5, Hinman Box 7251, NH 03756 Lebanon, NH USA; 2 Department of Surgery, University of Vermont Cancer Center, University of Vermont, Burlington, VT USA; 3 School of Medicine, University of North Carolina at Chapel Hill, Chapel Hill, NC USA; 4 Departments of Medicine, and Epidemiology and Biostatistics, University of California, San Francisco, CA USA; 5 Department of Public Health Sciences, University of California, Davis, CA USA; 6 Kaiser Permanente Washington Health Research Institute, Seattle, WA USA; 7 Department of Population Health Science, Huntsman Cancer Institute, University of Utah, Salt Lake City, UT USA; 8 Radiology Department, Dartmouth Health and Geisel School of Medicine at Dartmouth Lebanon, Lebanon, NH USA; 9 Dartmouth Cancer Center, Geisel School of Medicine at Dartmouth, Lebanon, NH USA

**Keywords:** Breast density, Breast cancer, Cancer screening, Cancer treatment, Patient-reported outcomes, BCSC

## Abstract

**Background:**

Following a breast cancer diagnosis, it is uncertain whether women’s breast density knowledge influences their willingness to undergo pre-operative imaging to detect additional cancer in their breasts. We evaluated women’s breast density knowledge and their willingness to delay treatment for pre-operative testing.

**Methods:**

We surveyed women identified in the Breast Cancer Surveillance Consortium aged ≥ 18 years, with first breast cancer diagnosed within the prior 6–18 months, who had at least one breast density measurement within the 5 years prior to their diagnosis. We assessed women’s breast density knowledge and correlates of willingness to delay treatment for 6 or more weeks for pre-operative imaging via logistic regression.

**Results:**

Survey participation was 28.3% (969/3,430). Seventy-two percent (469/647) of women with dense and 11% (34/322) with non-dense breasts correctly knew their density (*p* < 0.001); 69% (665/969) of all women knew dense breasts make it harder to detect cancers on a mammogram; and 29% (285/969) were willing to delay treatment ≥ 6 weeks to undergo pre-operative imaging. Willingness to delay treatment did not differ by self-reported density (OR:0.99 for non-dense vs. dense; 95%CI: 0.50–1.96). Treatment with chemotherapy was associated with less willingness to delay treatment (OR:0.67; 95%CI: 0.46–0.96). Having previously delayed breast cancer treatment more than 3 months was associated with an increased willingness to delay treatment for pre-operative imaging (OR:2.18; 95%CI: 1.26–3.77).

**Conclusions:**

Understanding of personal breast density was not associated with willingness to delay treatment 6 or more weeks for pre-operative imaging, but aspects of a woman’s treatment experience were.

**ClinicalTrials.gov:**

NCT02980848 registered December 2, 2016.

**Supplementary Information:**

The online version contains supplementary material available at 10.1186/s13058-024-01820-x.

## Background

Cancer treatment has improved over the last few decades resulting in more complex treatment planning and decision making [1]. However, breast cancer treatment delay can lead to poor health outcomes including decreased survival [2]. If pre-operative imaging, such as ultrasound, is performed there is a waiting period for results that may inform treatment planning but also may delay treatment. Clinically acceptable time from diagnosis to treatment varies and is dependent on the natural history of disease [3–6]. Balancing the benefits and harms of breast cancer treatment delay is dependent on a woman’s clinical scenario including her cancer type and stage, and her treatment preferences [6, 7]. 

About 40% of women in the United States (US) have mammographically dense breasts, hence forth referred to as dense breasts [8]. Breast density is a known independent risk factor for breast cancer, and dense breast tissue has a masking effect that decreases the sensitivity of mammography [9]. This has received much attention from lobbyists and policy makers over the last decade resulting in breast density notification laws in 38 states and the District of Columbia, and a national density notification law passed in 2019 with recently released implementation guidelines requiring all women receive information about if they have dense or non-dense breast tissue after mammography [10, 11]. 

The American College of Radiology practice guidelines suggest clinicians and women consider MRI to evaluate the extent of disease in the ipsilateral breast and screen for additional cancer in the contralateral breast in women with invasive cancer or ductal carcinoma in situ (DCIS) [12]. It is unclear to what extent women with dense breasts may be willing to delay treatment for their breast cancer in order to receive additional testing to detect additional cancer or the degree to which they are aware of their breast density and its implications. Women’s breast density knowledge has generally been captured through survey work and includes assumptions that if women report that they know their breast density or accurately define breast density, then they are correct in their knowledge of their personal breast density [13, 14]. Through our previous work comparing self-reported and clinical density measurements we found that women of screening age who have not had breast cancer did not accurately know their breast density [15], and there is evidence that women do not have a strong understanding of the association between breast density and breast cancer risk [16]. Yet, little is known about how increasing exposure to breast density information affects accuracy of knowledge of personal breast density or preferences towards pre-operative imaging and treatment in women diagnosed with breast cancer. In this study we surveyed women recently diagnosed with breast cancer and assessed the accuracy of their breast density knowledge, their willingness to undergo pre-operative imaging that would delay treatment but identify any additional cancer present in their breasts, and correlates of willingness to delay treatment 6 or more weeks. Study findings may be informative in tailoring clinical communications and treatment planning for women recently diagnosed with breast cancer.

## Methods

### Setting

The Breast Cancer Surveillance Consortium (BCSC) is a network of breast imaging registries that link their local radiology facility data with state or regional tumor registries and pathology databases [17]. Detailed data including breast imaging collected during screening, resulting BI-RADS assessments and breast density determination made by interpreting radiologists, pathology, clinical history, and sociodemographics are pooled at a BCSC Statistical Coordinating Center. BCSC registries included in this study were: the Carolina Mammography Registry, Sacramento Area Breast Imaging Registry, San Francisco Mammography Registry, Vermont Breast Cancer Surveillance System, Metro Chicago Breast Cancer Registry, New Hampshire Mammography Network, and Kaiser Permanente Washington.

### Participants

We invited women 18 years or older who were diagnosed with an incident breast cancer (stages 0-III) within the prior 6–18 months.

### Recruitment

From December 2017 through January 2020 eligible participants were identified by BCSC registries and invited to participate in a web-based survey via letters sent through the US postal service. A $2 bill incentive was included with mailed invitations from six registries. Invitees had the option to request a paper survey at all but one registry. One registry offered an option to complete the survey via telephone. Participants were assigned a unique identification number and access code to complete the survey on a secure web-portal. Registry staff used a secure internet application to generate each identification number and access code at the time of data import of eligible women from registry databases. Up to three reminder postcards to complete the survey were sent to eligible participants. Participants from five registries were entered for a chance to win a $100 gift card with one winner per registry. Each institution received approval from their institutional review board.

### Survey content and clinical measures

Survey content was informed by input from patient partners, a patient advisory board, and results from previously conducted focus groups [14]. 

#### Patient characteristics

Women self-reported sociodemographic characteristics and information about their previous experience with breast cancer screening and treatment including age, race/ethnicity, education, family history of breast cancer, insurance status, breast cancer mode of detection, prior screening or diagnostic MRI, breast cancer surgery type, time from diagnosis to first surgery, and additional treatment after first breast cancer surgery. Knowledge and feelings about the implications of breast density were also reported via the survey. The American Joint Committee on Cancer (AJCC) diagnostic stage of breast cancer [18], urban/rural residence, and clinical breast density were obtained through BCSC registry data. State breast density notification law was categorized by location where breast cancer screening and treatment was received.

#### Breast density knowledge and implications

To assess knowledge of personal breast density we asked, “Have you ever been told by a health care provider that you have dense breasts?” To assess knowledge of the implications of breast density we asked, “What do you think having dense breasts means?” Response categories are shown in Table 1.

**Table 1:** Self-Report of Breast Density, Density Knowledge, and Worry About Future Breast Cancer by BI-RADS^ Breast Density in 5 Years Prior to Survey

	Participants Overall	BI-RADS Breast Density Measured on Exams in Prior 5 Years		*p*-value
		Never Dense^*^	Ever Dense^*^	
	*n* = 969 (%)	*n* *=* 322 (%)	*n* = 647 (%)	
**Have you ever been told by a health care provider that you have “dense breasts”?**				< 0.001
Was told dense breasts	582 (60)	113 (35)	469 (72)	
Was told do not have dense breasts	46 (5)	34 (11)	12 (2)	
Not been told dense breasts or not	164 (17)	80 (25)	84 (13)	
I do not know/no response	177 (18)	95 (30)	82 (13)	
**Knew whether had dense breasts or not before cancer diagnosis**				0.084
Yes	516 (53)	113 (35)	403 (62)	
No	108 (11)	32 (10)	76 (12)	
No response	4 (< 1)	2(< 1)	2(< 1)	
Not asked^†^	341 (35)	175 (54)	166 (26)	
What do you think having dense breasts means?^**^				
Hard to see cancers on a mammogram	665 (69)	184 (58)	481 (75)	< 0.001
It is more difficult for a doctor to read a mammogram	502 (52)	130 (41)	372 (58)	< 0.001
A mammogram often has to be repeated to get a better picture	462 (48)	135 (43)	327 (51)	0.020
Other imaging tests are needed	485 (50)	135 (43)	350 (54)	0.001
More likely to get breast cancer	189 (20)	35 (11)	154 (24)	< 0.001
Don’t know what it means	149 (16)	73 (23)	76 (12)	< 0.001
**Would or does having dense breasts make you worry about future breast cancer?**				< 0.001
Yes	323 (33)	81 (25)	242 (37)	
No	388 (40)	122 (38)	266 (41)	
I don’t know/No response	258 (27)	119 (37)	139 (21)	

^ Breast Imaging Reporting and Data

*Never-dense = Did not receive a Breast Imaging-Reporting and Data System (BI-RADS) c or d in prior 5 years; Ever-dense = Received a BI-RADS c or d in the prior 5 years

† Not asked if responded not told, didn’t know, or didn’t respond to “Have you ever been told by a health care provider that you have “dense breasts”?”

**Not mutually exclusive categories

#### Clinical breast density

Five years of breast density measurements prior to the survey date were obtained from BCSC registries. Breast Imaging Reporting and Data (BI-RADS) breast density was classified by the interpreting radiologist as: a = almost entirely fat, b = scattered fibroglandular densities, c = heterogeneously dense, or d = extremely dense. BI-RADS a and b were considered “non-dense” breasts and BI-RADS c and d were considered “dense” breasts. Each survey participant’s breast density was classified as either “never-dense” or “ever-dense” based on whether BI-RADS c or d were ever observed in the five years prior to the survey or not.

#### State breast density notification law

All registries were assigned a notification category depending on state density reporting laws during our survey period. All states but one had laws in place during our survey period. One state assigned to the “notify women with dense and non-dense breasts” category implemented their law six months after the date of survey initiation. However, registry facilities from that state reported their health system had already implemented breast density notification to all women before survey initiation. One state in the “notify women with dense breasts only” category implemented their law 10 months after survey initiation, but the partnering health systems began notifying women with dense breasts before survey initiation as part of preparation to meet the law’s reporting requirements.

#### Willingness to delay breast cancer treatment 6 or more weeks

Following a series of questions that asked participants to consider their recent breast cancer diagnostic and treatment experiences, women were presented with the scenario “Some women have breast cancer in more than one place in their breasts that may not be seen on a mammogram.” The women were then asked “imagine there is a test that could tell you if there is any other cancer in your breasts. Would you choose to have the test if having it would delay cancer treatment by…” Each response category was asked as a separate question with time periods including 2 weeks, 4 weeks, 6 weeks, and 8 weeks. For each time period participants could answer either “yes, no, or I don’t know.” For our primary analysis we categorized willingness to delay treatment at each time period as having said yes at that time period and the previous time period(s). A response of “I don’t know” or no answer was classified as not indicating a willingness to delay treatment. Willingness to delay treatment 6 or more weeks included women willing to delay at-least 6 weeks. All other responses were categorized as not willing to delay 6 or more weeks. We chose to assess willingness to delay treatment 6 or more weeks because the majority of patients with a breast cancer diagnosis in the US have an existing wait time of approximately 4 weeks or fewer [19, 20], and multiple healthcare systems around the world have set 31 days as a maximal acceptable wait time from discussing a diagnosis with a patient to first treatment initiation [21]. We aimed to assess characteristics of women willing to wait longer than the average wait time.

### Statistical analysis

We compared participant characteristics by breast density (ever vs. never-dense over the prior five years) and outcome response patterns using Chi-square tests. Multivariable logistic regression was used to identify the correlates of being willing to delay treatment 6 or more weeks for additional testing following a breast cancer diagnosis. Model selection was performed using the backward stepwise method with the likelihood ratio test which sequentially enters the most significant variable with *p* ≤ 0.10, then after each entered variable removes variables not maintaining a *p* ≤ 0.05 level of significance. We tested the categorical variables age, race/ethnicity, education, urban/rural residence, insurance status, family history of breast cancer, breast cancer mode of detection, AJCC diagnostic stage, state breast density notification law, prior screening or diagnostic MRI, time from diagnosis to first surgery, breast cancer surgery type, and additional treatment after first breast cancer surgery. Self-reported breast density was forced into the model. We report odds ratios and Wald 95% confidence intervals. Analyses were performed using Stata 17.0.

Sensitivity analyses were undertaken to determine if the correlates of delay changed when we classified the dependent variable differently based on participant response patterns in two ways: (1) we excluded participants who did not provide responses for all time points, or (2) we excluded timepoints when a participant did not provide response. For example, if a participant gave a preference for 2 weeks but not 4 weeks, we included their preference for 2 weeks but not 4 weeks.

## Results

A total of 28.3% (969/3,430) of invited women participated and were confirmed eligible. Our sample was highly educated with 88% (850/969) having received some level of college education. 87% (829/969) identified as White, non-Hispanic, 90% (873/969) lived in an urban location, 67% (647/969) had dense breasts reported from a mammogram in the past 5 years, and 93% (905/969) lived in a state with breast density reporting regulations (Table 2). 46% (445/969) had undergone breast MRI, 24% (228/969) reported waiting less than 1-month from their diagnosis to treatment, 90% (873/969) had early-stage breast cancer (stages 0-II) within the last 6–18 months, and 98% had surgery (949/969) for their cancer (Table 3). About half (178/322) of women with never-dense breasts lived in a state that reports breast density to both women with dense and non-dense breasts (Table 2), but 35% (113/322) of women with never-dense breasts reported being told they have dense breasts (Table 1) and only 11% (34/322) reported being told they have non-dense breasts. In contrast, 72% (469/647) of women with ever-dense breasts reported being told they have dense breasts (*p* < 0.001). Three-quarters (481/647) of women with ever-dense and 58% (184/322) with never-dense breasts knew that dense breasts makes it hard to see cancers on a mammogram (*p* < 0.001). In contrast, only 24% (154/647) of women with ever-dense and 11% (35/322) with never-dense breasts knew that having dense breasts results in someone being more likely to get breast cancer (*p* < 0.001). Half (485/969) of all women knew other imaging tests may be needed, and 33% (323/969) reported that breast density does or would make them worry about future breast cancer.

**Table 2:** Demographic characteristics by BI-RADS^ breast density measured on mammography exams in 5 years prior to survey and categorized as never dense or ever dense

Characteristics	Participants Overall	BI-RADS Breast Density Measured on Exams in Prior 5 Years		*p*-value
		Never Dense^*^	Ever Dense^*^	
	*n* = 969 (%)	*n* = 322 (%)	*n* = 647 (%)	
**Age, years**				< 0.001
18–49	120 (12)	18 (6)	102 (16)	
50–64	390 (41)	112 (35)	278 (43)	
65–74	353 (37)	146 (46)	207 (32)	
75 or older	99 (10)	40 (13)	59 (9)	
**Race and Ethnicity**				0.10
Asian non-Hispanic	33 (3)	6 (2)	27 (4)	
Black non-Hispanic	44 (5)	11 (4)	33 (5)	
Hispanic/Latina	36 (4)	15 (5)	21 (3)	
White non-Hispanic	829 (87)	276 (88)	553 (87)	
Other or multiracial, non-Hispanic	9 (1)	5 (2)	4 (1)	
**Work Status**				0.001
Working full time	331 (35)	87 (27)	244 (38)	
Working part time	150 (16)	48 (15)	102 (16)	
Retired	404 (42)	161 (51)	243 (38)	
Unemployed/Disabled	71 (7)	22 (7)	49 (8)	
**Education**				< 0.001
High school or less	111 (12)	54 (17)	57 (9)	
Some college	293 (30)	115 (36)	178 (28)	
4-year college	206 (21)	49 (15)	157 (24)	
> 4-year college	351 (37)	99 (31)	252 (39)	
**Urban/Rural Residence**				0.186
Urban	873 (90)	282 (88)	591 (91)	
Rural	96 (10)	40 (12)	56 (9)	
**Insurance Status**				< 0.001
Medicare	427 (44)	179 (56)	248 (38)	
Medicaid/uninsured	30 (3)	11 (3)	19 (3)	
Private	507 (53)	128 (40)	379 (59)	
**Family History of Breast Cancer**				0.117
Yes	285 (30)	83 (27)	202 (32)	
No	667 (70)	229 (73)	438 (68)	
**State Breast Density Notification Law**				0.30
Notifies for women with dense and non-dense breasts	507 (52)	178 (55)	329 (51)	
Notifies for women with dense breasts only	398 (41)	121 (38)	277 (43)	
No notification mandate	64 (7)	23 (7)	41 (6)	

^ Breast Imaging Reporting and Data

*Never-dense = Did not receive a Breast Imaging-Reporting and Data System (BI-RADS) c or d in prior 5 years; Ever-dense = Received a BI-RADS c or d in the prior 5 years

**Table 3:** Clinical characteristics by BI-RADS^ breast density measured on mammography exams in 5 years prior to survey and categorized as never or ever dense

Characteristics	Participants Overall	BI-RADS Breast Density Measured on Exams in Prior 5 Years		*p*-value
		Never Dense^*^	Ever Dense^*^	
	*n* = 969 (%)	*n* = 322 (%)	*n* = 647 (%)	
**Breast Cancer Mode of Detection**				0.008
Self-Detection	238 (25)	69 (21)	169 (26)	
Screening Mammogram	654 (67)	236 (73)	418 (65)	
Other Clinical Detection	73 (8)	16 (5)	57 (9)	
**AJCC Stage** ^†^				0.020
DCIS	186 (19)	55 (17)	131 (20)	
I	502 (52)	183 (58)	319 (50)	
II	185 (19)	51 (16)	134 (21)	
III	52 (5)	12 (4)	40 (6)	
**Prior Screening or Diagnostic MRI**				0.006
Yes	445 (46)	127 (40)	318 (49)	
No	521 (54)	192 (60)	329 (51)	
**Time From Diagnosis to First Surgery**				0.464
< 1 month	228 (24)	77 (25)	151 (24)	
1–2 months	441 (47)	146 (47)	295 (47)	
2–3 months	151 (16)	55 (18)	96 (15)	
> 3 months	127 (13)	35 (11)	92 (15)	
**First Cancer Surgery Type**				< 0.001
Lumpectomy	701 (72)	253 (79)	448 (69)	
Mastectomy (or double)	248 (26)	60 (19)	188 (29)	
No surgical treatment	18 (2)	7 (2)	11 (2)	
**Additional Treatment After First Surgery**				
Mastectomy (unilateral or double)	62 (6)	22 (7)	40 (6)	0.669
Radiation Therapy	609 (63)	202 (63)	407 (63)	0.462
Hormone Therapy	457 (47)	147 (46)	310 (48)	0.403
Chemotherapy	223 (23)	76 (24)	147 (23)	0.432
Breast reconstruction	137 (14)	32 (10)	105 (16)	0.016
No additional treatment	97 (10)	35 (10)	62 (10)	0.370

^ Breast Imaging Reporting and Data

*Never-dense = Did not receive a Breast Imaging-Reporting and Data System (BI-RADS) c or d in prior 5 years; Ever-dense = Received a BI-RADS c or d in the prior 5 years

† American Joint Committee on Cancer

**Fig. 1:**
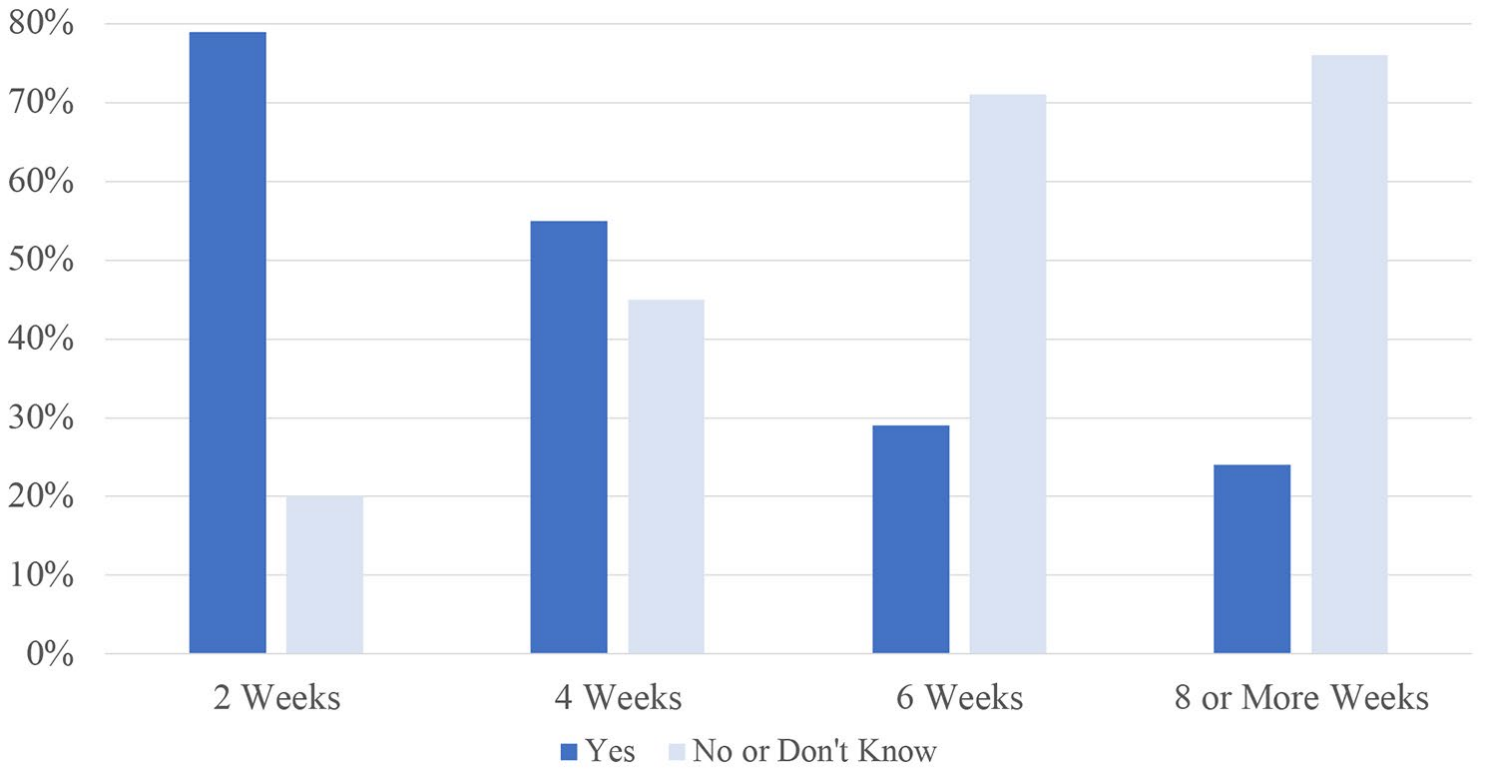
Length of time willing to delay breast cancer treatment for additional testing (*n* = 969)

Following their breast cancer diagnosis 79% (769/969) reported a willingness to delay treatment 2 weeks for pre-operative imaging that could detect additional cancer in their breasts (Fig. 1). More women were willing to delay treatment 4 weeks for additional testing than not (55%; 529/969 vs. 45%; 440/969), but this pattern changed at 6 weeks when not as many women were willing to delay compared to women who were not willing to delay treatment by 6 weeks (29%; 285/969 vs. 71%; 684/969).

Willingness to delay breast cancer treatment 6 or more weeks for pre-operative imaging did not differ by self-report of breast density (odds ratio [OR]:0.99; 95% confidence interval [CI]: 0.50–1.96 for non-dense vs. dense breasts; OR:0.79; 95%CI: 0.57–1.08 for “did not know” vs. dense breasts; Table 4). Waiting more than 3 months for treatment of their breast cancer was associated with an increased odds of being willing to delay treatment by 6 or more weeks (OR:2.18; 95%CI: 1.26–3.77). Receipt of chemotherapy was associated with decreased odds of willingness to delay treatment (OR:0.67; 95%CI: 0.46–0.96) and being retired vs. working full-time was associated with decreased odds of willingness to delay treatment (OR:0.69; 95%CI: 0.49–0.97). Factors not significantly associated with willingness to delay treatment included age, race/ethnicity, education, urban/rural residence, insurance status, family history of breast cancer, breast cancer mode of detection, AJCC diagnostic stage, state breast density reporting law, prior screening or diagnostic MRI, breast cancer first surgery type, and additional treatment after first cancer surgery (except for chemotherapy). Unadjusted odds ratios for these can be found in the Appendix (supplementary Table 1).

**Table 4:** Odds of being willing to delay breast cancer treatment 6 or more weeks for additional testing (*n* = 911)^^^

Characteristics	Unadjusted	Adjusted
	OR^*^ (95% CI)^**^	OR^*^ (95% CI)^**^
**Self-Report Dense Breasts**		
Yes	1 [Reference]	1 [Reference]
No	0.94 (0.49–1.80)	0.99 (0.50–1.96)
Did not know/Was not told	0.73 (0.54–0.98)	0.79 (0.57–1.08)
**Work Status**		
Working fulltime	1 [Reference]	1 [Reference]
Working part time	1.71 (1.15–2.56)	1.21 (0.80–1.83)
Retired	0.69 (0.50–0.95)	0.69 (0.49–0.97)
Unemployed/Disabled	0.64 (0.35–1.16)	0.56 (0.29–1.07)
**Time From Diagnosis to First Surgery**		
< 1 month	1 [Reference]	1 [Reference]
1–2 months	0.93 (0.56–1.55)	0.96 (0.57–1.63)
2–3 months	1.42 (0.91–2.23)	1.37 (0.85–2.20)
> 3 months	2.31 (1.38–3.87)	2.18 (1.26–3.77)
**Additional Treatment After First Surgery** ^†^		
Mastectomy (unilateral or double)	0.82 (0.46–1.48)	-
Radiation Therapy	0.89 (0.67–1.19)	-
Hormone Therapy	0.89 (0.68–1.18)	-
Chemotherapy	0.71 (0.50-1.00)	0.67 (0.46–0.96)
Breast reconstruction	0.91 (0.61–1.36)	-
No additional treatment	1.05 (0.84–1.31)	-

^ age, race/ethnicity, education, urban/rural residence, insurance status, family history of breast cancer, breast cancer mode of detection, AJCC diagnostic stage, state breast density notification law, prior screening or diagnostic MRI, breast cancer first surgery type, and additional treatment after first cancer surgery (except for chemotherapy) were tested but did not significantly contribute to the model

* Odds Ratio

** Confidence Interval

† not having the treatment is the reference for each category

Only 9% (90/969) of women did not provide an answer for at-least one treatment delay time point. In a sensitivity analysis, categorization of these women did not impact our regression results when we included a woman’s preference at each time point regardless of responses at other timepoints. When we excluded those who did not provide responses for all time points, results were minimally impacted with employment status “retired” no longer significantly associated with willingness to delay treatment (Supplementary Tables 2 and 3).

## Discussion

Women are increasingly exposed to breast density information yet breast density knowledge and its influence on attitudes toward pre-operative breast cancer imaging after a breast cancer diagnosis are poorly understood. Among a population of women who recently had a breast cancer diagnosis, we found that more than one-third of women with never-dense breasts reported being told they have dense breasts. While there is room for clinical error in communication, these women never had a dense breast measurement in the past 5 years in a facility in our study. This implies women may be misunderstanding breast density information shared with them, or that clinicians are not following up with all women to ensure they understand their individual risk factors for breast cancer. It is reasonable to expect that women who had a breast cancer in the last 6–18 months have more knowledge of their breast density given increased interactions with their care teams regarding their breast health. Yet, over a quarter of women with dense breasts reported not being told or not knowing if they were told their density. These findings are like our previous work examining breast density knowledge in a screening population of women with no personal history of breast cancer [15]. 

We found that women’s self-reported breast density was not associated with willingness to delay breast cancer treatment 6 or more weeks for pre-operative imaging that could detect additional cancer in their breasts. However, only about half of the women in our sample understood most of the implications of breast density including that other imaging tests beyond mammography may be needed to detect the full extent of malignancy. This indicates that about half of women do not fully understand what breast density means in relation to pre-operative imaging during the diagnostic workup period.

Why so many women did not accurately know their breast density or implications of breast density is not clear. In our sample, 45% of women with clinically non-dense breasts did not live in states where breast density notifications were required for women with non-dense breasts. For those who lived where they would have received a density notification, additional information about density implications may not have been included in their notification. There may not have been much incentive for clinicians to discuss density and its implications further with these women given that their mammography would generally have been considered sufficient imaging. However, two-thirds of women in our study did have a dense breast measurement in the past 5 years and 94% of them lived in a state with mandatory density notifications. This indicates that information about breast density in their notifications was not always understood and that breast density was not further discussed with their providers in a way that resulted in most women accurately retaining that information. Women may not ask providers about the breast density information in their notifications because they are worried or overwhelmed by their breast cancer diagnosis [22, 23]. 

Studies have shown that breast ultrasound and MRI result in high rates of false-positives although a multicenter breast screening trial found that high false-positive rates diminished over time as radiologists and clinical staff gained experience with adjunctive screening using ultrasound [24]. Additionally, a 2018 study found that false-positives from MRI had a higher probability of being due to high-risk atypical proliferative changes [25]. Breast MRI has been associated with a potential increase in the rate of overdiagnosis [26], but MRI of the contralateral breast yields a high and reliable negative predictive value that may be helpful during the treatment planning period [27]. Thus, there is a tradeoff and a woman’s choice should be elicited when pre-operative imaging is clinically appropriate.

Pre-operative imaging does lengthen the time from diagnosis to first cancer treatment [2], and studies have had mixed findings on the impact of treatment delay on patient outcomes [28]. One study found that delay greater than 90 days for patients with non-invasive breast cancer did not lead to decreased survival [4]. A study using SEER Medicare claims found disease specific survival decreased by a relative 24% per month of delay [29]. A commonly accepted time to treatment following diagnosis is one month [2, 21]. While some countries such as The United Kingdom have this as a set guideline [21], the United States does not have a commonly agreed upon time standard given treatment initiation is disease and woman specific.

More than half (55%) of women in our sample were willing to delay treatment 4 weeks and almost a third of women were willing to delay treatment for 6 weeks. Placing more value on a test’s potential to detect cancer versus harms such as false-positives has been previously documented [30, 31]. Getting a breast cancer diagnosis is a stressful event in a woman’s life and may impact the ability to engage in decision-making [32]. Our prior work demonstrated that most women with a recent breast cancer diagnosis had some level of cancer worry and treatment decisional regret [33]. Additionally, a woman feeling overwhelmed following a cancer diagnosis or feeling that she does not have enough information and support can lead to the sense that she has inappropriate input in the treatment decision making process [34]. This emphasizes a need for supported and tailored clinical communications to ensure a woman is equipped to engage appropriately in the treatment decision making process.

Women’s preferences may be impacted by their experience with breast cancer. Those reporting waiting more than 3 months from diagnosis to treatment for their breast cancer had increased odds of being willing to delay treatment by 6 or more weeks for additional testing. If a woman underwent chemotherapy treatment, she was less willing to delay treatment for adjunct pre-operative imaging. The larger a woman’s tumor size or the later her stage of cancer, the more likely it is that chemotherapy will be a recommended part of her treatment plan [35], and women may assign a lower value to pre-operative imaging that would delay treatment if they believe imaging missed their cancer at an earlier stage.

There are other reasons for delayed treatment. Multiple studies found Black women have longer times to treatment than White women [36, 37], but we did not find any difference in willingness to delay by race or other sociodemographic factors other than employment. The cost of pre-operative imaging is not always covered by insurance. However, through previous work we did not find that cost was an important factor in the cancer diagnostic workup and treatment decision making [33]. 

Our study had both strengths and limitations. We assessed a national sample representing multiple healthcare systems and practice norms, but our study sample was predominately white race and highly educated limiting the potential generalizability of study findings. Women in our sample who have non-dense breasts may have had a mammogram indicating “dense breasts” prior to the five-year period included in our clinical data or from a non-BCSC facility. We did not ask about willingness to delay treatment beyond 8 weeks, and thus, cannot distinguish the number willing to wait only 8 weeks from those willing to delay treatment longer for additional testing. However, the change in preferences between willingness to wait 6 and 8 or more weeks was minimal.

We found that self-reported extent of breast density is not associated with willingness to delay treatment 6 or more weeks for pre-operative imaging, but components of a woman’s treatment experience, including waiting more than 3 months for treatment of their breast cancer and receipt of chemotherapy, increased and decreased respectively willingness to delay treatment for pre-operative imaging. This highlights the importance of clinical communication during the treatment planning period, which should consider women’s diagnostic and treatment experiences with breast cancer. There are multiple potential benefits and harms to delaying breast cancer treatment for pre-operative imaging, and patient preferences should be incorporated into discussions about these tradeoffs during the treatment decision making process.

### Electronic supplementary material

Below is the link to the electronic supplementary material.

Supplementary Material 1

## Data Availability

The data underlying this article will be shared on reasonable request to the corresponding author, with appropriate regulatory approvals.

## References

[1] Bleicher RJ, Ruth K, Sigurdson ER, Ross E, Wong YN, Patel SA (2012). Preoperative delays in the US Medicare population with breast cancer. J Clin Oncol.

[2] Bleicher RJ (2018). Timing and delays in breast Cancer evaluation and treatment. Ann Surg Oncol.

[3] Chavez-MacGregor M, Clarke CA, Lichtensztajn DY, Giordano SH (2016). Delayed initiation of adjuvant chemotherapy among patients with breast Cancer. JAMA Oncol.

[4] Ho PJ, Cook AR, Binte Mohamed Ri NK, Liu J, Li J, Hartman M (2020). Impact of delayed treatment in women diagnosed with breast cancer: a population-based study. Cancer Med.

[5] Mateo AM, Mazor AM, Obeid E, Sigurdson ER, DeMora L, Handorf EA, et al. Time to surgery and the impact of delay on triple negative breast cancers and other phenotypes. American Society of Clinical Oncology; 2018. 10.1245/s10434-019-08050-y PMC7145740 31712923

[6] Polverini AC, Nelson RA, Marcinkowski E, Jones VC, Lai L, Mortimer JE (2016). Time to treatment: measuring quality breast Cancer Care. Ann Surg Oncol.

[7] Westendorp J, Evers AWM, Stouthard JML, Budding J, van der Wall E, Plum NMF (2022). Mind your words: oncologists’ communication that potentially harms patients with advanced cancer: a survey on patient perspectives. Cancer.

[8] Centers for Disease Control and Prevention. Dense Breast Inform https://www.cdc.gov/cancer/breast/basic_info/dense-breasts.htm#:~:text=The%20breasts%20are%20almost%20entirely,about%2040%25%20of%20women accessed August 22, 2023.

[9] National Cancer Institute. Dense Breasts: Answers to Commonly Asked Questions. https://www.cancer.gov/types/breast/breast-changes/dense-breasts#:~:text=However%2C%20dense%20breasts%20are%20a,ability%20to%20read%20a%20mammogram accessed August 22, 2023.

[10] densebreast-info.org. State Legislation Map. https://densebreast-info.org/legislative-information/state-legislation-map/ accessed September 1, 2022.

[11] *Mammography Quality Standards Act*, 2023.

[12] American College of Radiology. ACR Practice Parameter for the Performance of Contratenhanced Magnetic Rosonance Imaging (MRI) of the Breast. Available: https://www.acr.org/-/media/acr/files/practice-parameters/mr-contrast-breast.pdf

[13] Manning MA, Duric N, Littrup P, Bey-Knight L, Penner L, Albrecht TL (2013). Knowledge of breast density and awareness of related breast cancer risk. J Cancer Educ.

[14] Schifferdecker KE, Tosteson ANA, Kaplan C, Kerlikowske K, Buist DSM, Henderson LM (2020). Knowledge and perception of breast density, Screening Mammography, and Supplemental Screening: in search of informed. J Gen Intern Med.

[15] Smith RE, Sprague B, Henderson LM, Kerlikowske K, Miglioretti DL, Buist DSM (2022). Breast density knowledge in a screening Mammography Population exposed to density notification. J Am Coll Radiol.

[16] Beidler LB, Kressin NR, Wormwood JB, Battaglia TA, Slanetz PJ, Gunn CM (2023). Perceptions of breast Cancer risks among women receiving Mammograph Screening. JAMA Netw Open.

[17] Breast Cancer Survielliance Consortium. About the BCSC. accessed August 29, 2022 https://www.bcsc-research.org/about#:~:text=The%20Breast%20Cancer%20Surveillance%20Consortium,outcomes%20in%20the%20United%20States

[18] Edge SB (2010). AJCC cancer staging manual. Springer.

[19] Khorana AA, Tullio K, Elson P, Pennell NA, Grobmyer SR, Kalady MF (2019). Time to initial cancer treatment in the United States and association with survival over time: an observational study. PLoS ONE.

[20] Cone EB, Marchese M, Paciotti M, Nguyen DD, Nabi J, Cole AP (2020). Assessment of Time-to-Treatment Initiation and Survival in a cohort of patients with common cancers. JAMA Netw Open.

[21] United Kingdom Cancer Research. Cancer Waiting Times 2022. https://www.cancerresearchuk.org/about-cancer/cancer-in-general/cancer-waiting-times accessed August 25, 2022.

[22] Longo V, Abruzzese F, Miserocchi V, Carriero S, Gambaro AC, Saba L et al. Breast cancer and communication: monocentric experience of a self-assessment questionnaire. J Public Health Res. 2022;11(2). 10.4081/jphr.2022.2831 PMC8973206 35315263

[23] Gibbons A, Groarke A, Sweeney K (2016). Predicting general and cancer-related distress in women with newly diagnosed breast cancer. BMC Cancer.

[24] Weigert JM (2017). The Connecticut experiment; the third installment: 4 years of Screening women with dense breasts with bilateral Ultrasound. Breast J.

[25] Kuhl CK, Keulers A, Strobel K, Schneider H, Gaisa N, Schrading S (2018). Not all false positive diagnoses are equal: on the prognostic implications of false-positive diagnoses made in breast MRI versus in mammography / digital tomosynthesis screening. Breast Cancer Res.

[26] Wang SY, Long JB, Killelea BK, Evans SB, Roberts KB, Silber A (2016). Preoperative breast magnetic resonance imaging and contralateral breast cancer occurrence among older women with ductal carcinoma in situ. Breast Cancer Res Treat.

[27] Debruhl ND, Lee SJ, Mahoney MC, Hanna L, Tuite C, Gatsonis CA (2020). MRI evaluation of the contralateral breast in women with recently diagnosed breast Cancer: 2-Year follow-up. J Breast Imaging.

[28] Rutter CM, Kim JJ, Meester RGS, Sprague BL, Burger EA, Zauber AG (2018). Effect of time to diagnostic testing for breast, cervical, and Colorectal Cancer Screening abnormalities on Screening Efficacy: a modeling study. Cancer Epidemiol Biomarkers Prev.

[29] Bleicher RJ, Ruth K, Sigurdson ER, Beck JR, Ross E, Wong Y-N (2016). Time to surgery and breast Cancer survival in the United States. JAMA Oncol.

[30] Lewis CL, Kistler CE, Amick HR, Watson LC, Bynum DL, Walter LC (2006). Older adults’ attitudes about continuing cancer screening later in life: a pilot study interviewing residents of two continuing care communities. BMC Geriatr.

[31] Petrova D, Garcia-Retamero R, Cokely ET (2015). Understanding the harms and benefits of cancer screening: a model of factors that shape informed decision making. Med Decis Making.

[32] Reyna VF, Nelson WL, Han PK, Pignone MP (2015). Decision making and cancer. Am Psychol.

[33] Wernli KJ, Smith RE, Henderson LM, Zhao W, Durham DD, Schifferdecker K (2022). Decision quality and regret with treatment decisions in women with breast cancer: pre-operative breast MRI and breast density. Breast Cancer Res Treat.

[34] Livaudais JC, Franco R, Fei K, Bickell NA (2013). Breast cancer treatment decision-making: are we asking too much of patients?. J Gen Intern Med.

[35] National Comprehensive Cancer Network. NCCN Breast Cancer Guidelines. https://www.nccn.org/guidelines/guidelines-detail?category=1&id=1419 accessed October 20, 2022.

[36] Smith EC, Ziogas A, Anton-Culver H (2013). Delay in surgical treatment and survival after breast cancer diagnosis in young women by race/ethnicity. JAMA Surg.

[37] Reeder-Hayes KE, Mayer SE, Olshan AF, Wheeler SB, Carey LA, Tse CK (2019). Race and delays in breast cancer treatment across the care continuum in the Carolina breast Cancer Study. Cancer.

