# Supplementary material for: Breast density knowledge and willingness to delay treatment for pre-operative breast cancer imaging among women with a personal history of breast cancer
Source: Breast Cancer Res. 2024 Apr 29;26:73. doi: 10.1186/s13058-024-01820-x (PMC11057127; doi:10.1186/s13058-024-01820-x)
Supplement: Supplementary file 1 — Supplementary Material 1 [file 13058_2024_1820_MOESM1_ESM.docx]

**Appendix:**

**Supplemental Table 1. Odds of Being Willing to Delay Breast Cancer Treatment**

**6 or More Weeks for Additional Testing (n=911)**

|  | **Unadjusted** | **Adjusted** |
| --- | --- | --- |
| **Characteristics** | **OR^*^ (95% CI)^**^** | **OR^*^ (95% CI)^**^** |
| **Age, years** |  |  |
| 18-49 | 0.99 (0.62-1.56) | - |
| 50-64 | 1.29 (0.95-1.76) | - |
| 65-74 | 1 [Reference] | - |
| 75 or older | 0.52 (0.29-0.92) | - |
| **Race and Ethnicity** |  |  |
| Asian non-Hispanic | 1 [Reference] | - |
| Black non-Hispanic | 0.78 (0.28-2.22) | - |
| Hispanic/Latina | 0.64 (0.21-1.98) | - |
| White non-Hispanic | 1.16 (0.53-2.53) | - |
| Other or multiracial, non-Hispanic | 1.33 (0.27-6.50) | - |
| **Self-Report Dense Breasts** |  |  |
| Yes | 1 [Reference] | 1 [Reference] |
| No | 0.94 (0.49-1.80) | 0.99 (0.50 - 1.96) |
| Did not know/Was not told | 0.73 (0.54-0.98) | 0.79 (0.57 - 1.08) |
| **Work Status** |  |  |
| Working fulltime | 1 [Reference] | 1 [Reference] |
| Working part time | 1.71 (1.15-2.56) | 1.21 (0.80-1.83) |
| Retired | 0.69 (0.50-0.95) | 0.69 (0.49-0.97) |
| Unemployed/Disabled | 0.64 (0.35-1.16) | 0.56 (0.29-1.07) |
| **Education** |  |  |
| High school or less | 0.62 (0.38-1.02) | - |
| Some college | 0.76 (0.54-1.07) | - |
| 4 - year college | 1.10 (0.76-1.58) | - |
| > 4 year college | 1 [Reference] | - |
| **Urban/Rural Residence** |  |  |
| Urban | 1 [Reference] | - |
| Rural | 1.16 (0.74-1.83) | - |
| **Insurance Status** |  |  |
| Medicare | 1 [Reference] | - |
| Medicaid/uninsured | 0.88 (0.37-2.10) | - |
| Private | 1.43 (1.07-1.90) | - |
| **Family History of Breast Cancer** |  |  |
| Yes | 0.93 (0.69-1.27) | - |
| No | 1 [Reference] | - |
| **Breast Cancer Mode of Detection** |  |  |
| Self-Detection | 0.62 (0.44-0.88) | - |
| Screening Mammogram | 1 [Reference] | - |
| Other Clinical Detection | 0.85 (0.50-1.45) | - |
| **AJCC Stage**^†^ |  |  |
| DCIS | 1 [Reference] | - |
| I | 0.88 (0.63-1.24) | - |
| II | 0.70 (0.45-1.07) | - |
| III | 0.48 (0.23-1.02) | - |
| **State Breast Density Notification Law** |  |  |
| Notifies for women with dense and non-dense breasts | 1 [Reference] | - |
| Notifies for women with dense breasts only | 0.97 (0.72-1.29) | - |
| No notification mandate | 1.08 (0.62-1.90) | - |
| **Prior Screening or Diagnostic MRI** |  |  |
| Yes | 1.21 (0.95-1.53) | - |
| No | 1 [Reference] | - |
| **Time From Diagnosis to First Surgery** |  |  |
| <1 month | 1 [Reference] | 1 [Reference] |
| 1-2 months | 0.93 (0.56-1.55) | 0.96 (0.57-1.63) |
| 2-3 months | 1.42 (0.91-2.23) | 1.37 (0.85-2.20) |
| >3 months | 2.31 (1.38-3.87) | 2.18 (1.26-3.77) |
| **First Cancer Surgery Type** |  |  |
| Lumpectomy | 1 [Reference] | - |
| Mastectomy (or double) | 1.00 (0.72-1.37) | - |
| No surgical treatment | 0.92 (0.32-2.61) | - |
| **Additional Treatment After First Surgery^^^** |  |  |
| Mastectomy (unilateral or double) | 0.82 (0.46-1.48) | - |
| Radiation Therapy | 0.89 (0.67-1.19) | - |
| Hormone Therapy | 0.89 (0.68-1.18) | - |
| Chemotherapy | 0.71 (0.50-1.00) | 0.67 (0.46-0.96) |
| Breast reconstruction | 0.91 (0.61-1.36) | - |
| No additional treatment | 1.05 (0.84-1.31) | - |

† American Joint Committee on Cancer

* Odds Ratio

** Confidence Interval

^ not having the treatment is the reference for each category

**Supplemental Table 2. Odds of Being Willing to Delay Breast Cancer Treatment**

**6 or More Weeks for Additional Testing (n=907)^^^**

|  | **Unadjusted** | **Adjusted** |
| --- | --- | --- |
| **Characteristics** | **OR^*^ (95% CI)^**^** | **OR^*^ (95% CI)^**^** |
| **Self-Report Dense Breasts** |  |  |
| Yes | 1 [Reference] | 1 [Reference] |
| No | 0.94 (0.49-1.80) | 0.99 (0.50 - 1.96) |
| Did not know/Was not told | 0.73 (0.54-0.98) | 0.79 (0.57 - 1.08) |
| **Work Status** |  |  |
| Working fulltime | 1 [Reference] | 1 [Reference] |
| Working part time | 1.71 (1.15-2.56) | 1.21 (0.80-1.83) |
| Retired | 0.69 (0.50-0.95) | 0.69 (0.49-0.97) |
| Unemployed/Disabled | 0.64 (0.35-1.16) | 0.56 (0.29-1.07) |
| **Time From Diagnosis to First Surgery** |  |  |
| <1 month | 1 [Reference] | 1 [Reference] |
| 1-2 months | 0.93 (0.56-1.55) | 0.96 (0.57-1.63) |
| 2-3 months | 1.42 (0.91-2.23) | 1.37 (0.85-2.20) |
| >3 months | 2.31 (1.38-3.87) | 2.18 (1.26-3.77) |
| **Additional Treatment After First Surgery**^†^ |  |  |
| Mastectomy (unilateral or double) | 0.82 (0.46-1.48) | - |
| Radiation Therapy | 0.89 (0.67-1.19) | - |
| Hormone Therapy | 0.89 (0.68-1.18) | - |
| Chemotherapy | 0.71 (0.50-1.00) | 0.67 (0.46-0.96) |
| Breast reconstruction | 0.91 (0.61-1.36) | - |
| No additional treatment | 1.05 (0.84-1.31) | - |

^ Model includes woman’s preference at each time point regardless of responses at other timepoints. Age, race/ethnicity, education, urban/rural residence, insurance status, family history of breast cancer, breast cancer mode of detection, AJCC diagnostic stage, state breast density notification law, prior screening or diagnostic MRI, breast cancer first surgery type, and additional treatment after first cancer surgery (except for chemotherapy) were tested but did not significantly contribute to the model

* Odds Ratio

** Confidence Interval

† not having the treatment is the reference for each category

**Supplemental Table 3. Odds of Being Willing to Delay Breast Cancer Treatment**

**6 or More Weeks for Additional Testing (n=829)^^^**

| **Characteristics** | **OR^*^ (95% CI)^**^** | **OR^*^ (95% CI)^**^** |
| --- | --- | --- |
| **Self-Report Dense Breasts** |  |  |
| Yes | 1 [Reference] | 1 [Reference] |
| No | 0.94 (0.49-1.80) | 0.94 (0.47 - 1.89) |
| Did not know/Was not told | 0.73 (0.54-0.98) | 0.85 (0.62 - 1.18) |
| **Work Status** |  |  |
| Working fulltime | 1 [Reference] | 1 [Reference] |
| Working part time | 1.71 (1.15-2.56) | 1.19 (0.78-1.82) |
| Retired | 0.69 (0.50-0.95) | 0.75 (0.53-1.06) |
| Unemployed/Disabled | 0.64 (0.35-1.16) | 0.56 (0.29-1.07) |
| **Time From Diagnosis to First Surgery** |  |  |
| <1 month | 1 [Reference] | 1 [Reference] |
| 1-2 months | 0.93 (0.56-1.55) | 0.89 (0.52-1.53) |
| 2-3 months | 1.42 (0.91-2.23) | 1.31 (0.81-2.13) |
| >3 months | 2.31 (1.38-3.87) | 2.00 (1.14-3.50) |
| **Additional Treatment After First Surgery**^†^ |  |  |
| Mastectomy (unilateralor double) | 0.82 (0.46-1.48) | - |
| Radiation Therapy | 0.89 (0.67-1.19) | - |
| Hormone Therapy | 0.89 (0.68-1.18) | - |
| Chemotherapy | 0.71 (0.50-1.00) | 0.63 (0.43-0.91) |
| Breast reconstruction | 0.91 (0.61-1.36) | - |
| No additional treatment | 1.05 (0.84-1.31) | - |

^ Model excludes women that did not provide responses for all time points. Age, race/ethnicity, education, urban/rural residence, insurance status, family history of breast cancer, breast cancer mode of detection, AJCC diagnostic stage, state breast density notification law, prior screening or diagnostic MRI, breast cancer first surgery type, and additional treatment after first cancer surgery (except for chemotherapy) were tested but did not significantly contribute to the model

* Odds Ratio

** Confidence Interval

† not having the treatment is the reference for each category
